# Draft genome sequences of multidrug-resistant *Escherichia coli* and *Salmonella enterica* serovar Kentucky isolates from chicken samples in Bangladesh

**DOI:** 10.1128/mra.01429-25

**Published:** 2026-02-09

**Authors:** Bibi Amena, Mohimanul Islam, Tanvir Ahmad Nizami, Himel Barua, Paritosh Kumar Biswas

**Affiliations:** 1 Department of Microbiology and Veterinary Public Health, Faculty of Veterinary Medicine, Chattogram Veterinary and Animal Sciences University 130057, Chattogram, Bangladesh; University of Maryland Baltimore, Baltimore, Maryland, USA

**Keywords:** antimicrobial resistance, *Escherichia coli*, foodborne pathogen, genomics, non-typhoidal *Salmonella*, zoonosis

## Abstract

Pathogenic *Escherichia coli* and *Salmonella* threaten poultry production and human health through zoonotic transmission. Whole-genome sequencing of multidrug-resistant isolates from Bangladeshi poultry provides critical insights into antimicrobial resistance and population structure, supporting integrated One Health approaches to combat antimicrobial resistance.

## ANNOUNCEMENT

Poultry farming is crucial to Bangladesh’s food security and economy but faces threats from zoonotic bacterial pathogens such as *Escherichia coli* and *Salmonella*. Indiscriminate antimicrobial use in poultry has accelerated the emergence of multidrug-resistant strains, raising public health concerns through potential human transmission (1). However, genomic data on antimicrobial resistance mechanisms of these pathogens in Bangladesh remain limited. Between July 2023 and June 2024, a total of 271 *E. coli* were isolated from cloacal swabs of 280 chickens collected from poultry farms and live bird markets (LBMs) in Chattogram, Bangladesh. Additionally, 76 zoonotic/non-typhoidal *Salmonella* were isolated from 228 naturally pooled floor litter samples collected from the same LBMs. Cloacal swabs and 1 g of floor litter were pre-enriched overnight in 9 mL buffered peptone water (BD Difco, USA). *E. coli* was isolated using MacConkey agar (Oxoid, UK) and Eosin Methylene Blue agar (Oxoid, UK) and identified at genus level by *adk* gene PCR (2). For *Salmonella*, pre-enriched samples were cultured on Modified Semisolid Rappaport-Vassiliadis (MSRV) agar (Oxoid, UK) supplemented with novobiocin (Oxoid, UK), followed by isolation on Brilliant Green Agar (Oxoid, UK) and genus-level PCR confirmation using ST11–ST15 primers (3). All media were incubated aerobically at 37°C for 24 h, except MSRV at 42°C, unless otherwise specified. Antimicrobial susceptibility testing was conducted against nine antimicrobials from seven major classes, following CLSI 2024 (4) guidelines. Six multidrug-resistant isolates, three belonging to *E. coli* and three to *Salmonella,* were randomly selected for whole-genome sequencing.

Genomic DNA was extracted from the six selected isolates after re-culturing for 24 h on 5% bovine blood agar (Oxoid, UK) at 37°C, using the FavorPrep Tissue Genomic DNA Extraction Kit (Favorgen, Taiwan), and DNA quality and quantity were evaluated with a NanoDrop One spectrophotometer (Thermo Fisher Scientific, USA). DNA libraries were prepared using the Nextera XT Library Preparation Kit (Illumina Inc., USA), and sequencing was performed on an Illumina NextSeq 2000 platform at the Poultry Research and Training Centre, Chattogram Veterinary and Animal Sciences University, Bangladesh, generating 2 × 150 bp paired-end reads. The raw reads were assessed using FastQC version 0.12.1 (5), and low-quality bases/adapters were trimmed with Fastp version 0.23.4 (6). *De novo* assemblies were generated using Shovill version 1.1.0 (7) with SPAdes version 3.14.1 (8), and assembly quality was verified by QUAST version 5.2.0 (9). Taxonomic confirmation was made by using Kraken2 version 2.1.3 (10), with all analyses conducted using the Galaxy platform version 24.1 (https://usegalaxy.org/) (11). Antimicrobial resistance genes and plasmid replicons were identified using ResFinder version 4.7.2 (12, 13) and PlasmidFinder version 2.1 (≥90% identity and ≥60% coverage) (13, 14). All software was used with default parameters unless otherwise specified. Genome annotation was performed with the Prokaryotic Genome Annotation Pipeline (PGAP) version 6.8 (15) at the National Center for Biotechnology Information following sequence submission. Detailed genomic features are provided in Table 1. Sequence types and clonal complexes were assigned via the PubMLST database (16). The results revealed diverse antimicrobial resistance genes and plasmid profiles, providing primary genetic data resources to assess the potential acquisition of these genes by human pathogens through food-chain zoonotic transmission.

**TABLE 1:** Metadata of *E. coli* and *Salmonella* isolates included in this genomic study

Strain ID	BAE-1	BAE-2	BAE-3	BAS-1	BAS-2	BAS-3
Species	*E. coli*	*E. coli*	*E. coli*	*Salmonella enterica* serovar Kentucky	*S. enterica* serovar Kentucky	*S. enterica* serovar Kentucky
Sample	Chicken cloacal swab	Chicken cloacal swab	Chicken cloacal swab	Chicken floor litter	Chicken floor litter	Chicken floor litter
SRA accession no.	SRR30733615	SRR30733614	SRR30733613	SRR30733612	SRR30733611	SRR30733610
GenBank accession no.	JBHXXE000000000	JBHXXF000000000	JBHXXG000000000	JBHXXH000000000	JBHXXI000000000	JBHXXJ000000000
CC^*a*^	–^*e*^	–	–	56	56	56
ST^*b*^	1589^*c*^	2705^*c*^	1196^*c*^	198	198	198
Genome size (bp)	5,007,901	5,095,251	5,774,720	4,813,480	4,815,697	5,014,170
GC content (%)	50.58	50.41	50.61	52.16	52.16	51.92
No. of contigs	134	242	445	66	85	102
*N*_50_ (bp)	157,766	174,966	118,871	356,946	273,961	392,608
No. of reads	1,552,126	1,173,450	2,424,993	3,103,857	841,564	1,149,410
Coverage (×)	75	56	101	151	45	56
Genes (total), PGAP	4,938	5,102	6,033	4,727	4,742	4,930
Antibiogram result (disk diffusion method)^*d*^	R: AMP, CN, CRO, CIP, TE, SXT, NA S: ETP I: FEP	R: AMP, CN, ETP, CIP, TE, SXT, NA S: CRO, FEP	R: AMP, CN, CRO, CIP, TE, SXT, NA S: ETP, FEP	R: AMP, CN, CRO, ETP, CIP, TE, SXT, NA I: FEP	R: AMP, CN, CRO, ETP, CIP, TE, SXT, NA I: FEP	R: AMP, CN, CIP, TE, SXT, NA S: CRO, ETP, FEP
Antimicrobial resistance genes (ResFinder) (12, 13)	*aac(3)-IV, aadA5, aph(4)-Ia, blaCTX-M-65, dfrA17, floR, mph(A), sul1, sul2, tet(A*)	*aac(3)-IIa, aac(3)-IId, aadA1, aadA17, aph(3')-Ia, blaDHA-1, blaTEM-1B, bleO, dfrA14, dfrA17, lnu(F), mph(A), OqxA, OqxB, qnrB4, qnrS13, sul1, sul2, sul3, tet(A*)	*aac(3)-IId, aph(3')-Ia, aph(3'')-Ib, aph (6)-Id, blaDHA-1, blaTEM-1A, dfrA12, dfrA14, dfrA17, floR, fosA4, mph(A), qnrB4, sul1, sul2, sul3*	*aac(3)-Id, aac(6')-Iaa, aadA7, blaTEM-1B, sul1, tet(A*)	*aac(3)-Id, aac(6')-Iaa, aadA7, blaTEM-1B, sul1, tet(A*)	*aac(6')-Iaa, aac(3)-Id, aadA2, aadA7, aph(3')-Ia, aph(3'')-Ib, aph(6)-Id, blaTEM-1B, blaTEM-30, blaTEM-70, blaTEM-104, blaTEM-148, blaTEM-176, blaTEM-198, blaTEM-207, blaTEM-217, blaTEM-220, blaTEM-230, blaTEM-234, bleO, dfrA12, floR, mph(A), OqxA, OqxB, sul1, tet(A*)
Plasmids (PlasmidFinder) (13, 14)	Col(pHAD28), IncY	Col(MG828), IncFII, IncHI2, IncHI2A, IncN, IncX1, p0111	Col156, Col(MG828), IncB/O/K/Z, IncFIA(HI1), IncFIB(AP001918), IncFII, IncHI1A, IncHI1B(R27)	–	–	Col(pHAD28), IncHI2, IncHI2A

^*a*^ CC, clonal complex.

^*b*^ ST, sequence type.

^*c*^ Achtman scheme.

^*d*^ AMP, ampicillin; CN, gentamicin; CRO, ceftriaxone; ETP, ertapenem; FEP, cefepime; CIP, ciprofloxacin; TE, tetracycline; SXT, trimethoprim/sulfamethoxazole; and NA, nalidixic acid. R, resistant; S, sensitive; and I, intermediate.

^*e*^ “–” indicates that no data were identified.

## Data Availability

All six *E. coli* and *Salmonella* genome sequences are available under BioProject accession number PRJNA1162444, with individual accession numbers as provided in Table 1.
